# How sex affects the sinus rhythm heartbeat

**DOI:** 10.1016/j.ijcha.2023.101314

**Published:** 2023-11-27

**Authors:** Danny Veen, Corina Schram-Serban, Mathijs van Schie, Frank van Schaagen, Paul Knops, Maryam Kavousi, Yannick Taverne, Natasja M.S. de Groot

**Affiliations:** a Dept of Cardiology, Erasmus University Medical Center, Rotterdam, the Netherlands; b Dept of Cardio-Thoracic Surgery, Erasmus University Medical Center, Rotterdam, the Netherlands; c Dept of Epidemiology, Erasmus MC, University Medical Center, Rotterdam, the Netherlands; d Dept of Micro-electronics, Circuits and Systems, Faculty of Electrical Engineering, the Netherlands

**Keywords:** Sex differences, Atrial mapping, Conduction disorders, Electrophysiological properties

## Abstract

**Background:**

There is increasing awareness of sex-specific differences in epidemiology and pathophysiology of atrial fibrillation (AF). It is, however, unknown whether males and females differ in atrial electrophysiological properties during sinus rhythm (SR). The aim of this study was therefore to investigate sex-based (regional) differences in electrophysiological properties during SR of the right (RA) and left (LA) atrium including Bachmanns Bundle (BB) and pulmonary vein region (PVA).

**Methods:**

Intra-operative, high resolution mapping during SR was performed in 53 matched females with males (without a history of AF), to measure lines of conduction block (CB), continuous conduction delay and block (cCDCB), conduction velocities (CV), total atrial activation times (TAT), unipolar potential voltages and percentage of low voltage areas (LVA).

**Results:**

Compared to males, females have significantly 1) lower unipolar potential voltages and slower CV at both RA and BB, 2) more LVAs, CB and cCDCB lines and longer CB and cCDCB lines at the RA only (all P < 0.05).

**Conclusions:**

Electrophysiological properties of the atria during SR differ between males and females. These sex-based differences are particularly present at the RA and to a lesser degree at BB. In females, both the RA and BB contained more areas of conduction disorders and low voltage potentials. Future studies are required to investigate whether these areas play a role in sex-based differences in vulnerability to arrhythmias such as atrial fibrillation.

## Graphical abstract

**Figure:**
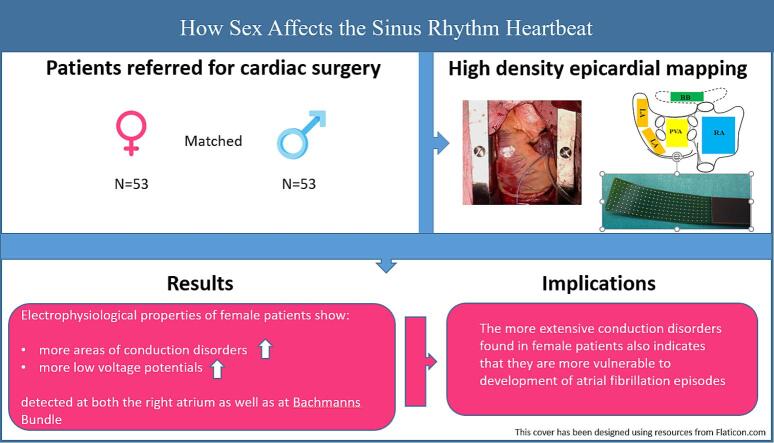

## 1 Introduction

There is increasing awareness of sex-specific differences in epidemiology and pathophysiology of cardiac arrhythmias. Atrial fibrillation (AF) for example, occurs more frequently in males than in females, irrespective of age [1], [2]. Female patients however, experience more often symptoms and have higher ventricular rates during AF episodes [1], [3], [4]. In addition, rhythm control therapy in females is less effective [5], [6] and they are more likely to develop sinus node dysfunction [1]. These observations suggest that females may have a more extensive arrhythmogenic substrate underlying AF [1], [6]. AF-related sex differences in the arrhythmogenic substrate were investigated and left atrial electrophysiological properties assessed in male and female patients were related with ablation outcome [7]. During coronary sinus pacing, conduction velocity (CV) was lower in female patients and they had a higher proportion of fractionated potentials and lower bipolar potentials voltages compared to male patients, which was related to the higher post-procedural AF recurrence rates observed in females [7]. This observation raises the question whether sex-based differences in atrial electrophysiological properties are only present when patients already have AF, or whether they also exist before AF develops.

However, at present, data on sex-specific differences in electrophysiological properties of the atria during sinus rhythm are limited to ECG features. Males have significantly longer P-wave durations compared to females [8], which may be caused by more extensive intra- and/or inter atrial conduction disorders [9]*.*

The aim of this study was therefore to investigate sex-specific, regional differences in atrial electrophysiological properties during SR by using high resolution mapping of the right (RA) and left atrium (LA) including Bachmanns Bundle (BB) and pulmonary vein area (PVA) in male and female patients (without a history of AF) undergoing cardiac surgery.

## 2 Methods

### 2.1 Study population

All patients were retrieved from an existing database containing patients who underwent a high-resolution mapping procedure during cardiac surgery. Males and female patients without a history of AF were matched based on underlying heart disease (and thus also surgical indications) age, left atrial dimension and presence of obesity (BMI > 30. Additionally we demonstrated in prior studies using similar high resolution mapping procedures that underlying heart diseases had no impact on conduction properties assessed during sinus rhythm.[10]).

This study was performed as part of two prospective observational projects (MEC 2010–054 and MEC 2014–393) [11], [12], [13]. Patients with prior ablation of atrial tachyarrhythmias, severe renal failure, an atrial pacing device or requiring inotropic support were excluded. Approval of both projects were granted by the local ethics committee of the Erasmus Medical Centre and adhere to the Declaration of Helsinki principles; written consent was obtained from participating patients before the surgical intervention.

### 2.2 Intra-Operative mapping procedure

High resolution mapping of the epicardium was performed prior to commencement to extra-corporal circulation, as previously described in detail [11], [12]. A bipolar wire was stitched to the epicardial wall and used a reference electrode, whereas a steel wire fixed to the subcutaneous tissue was used as an indifferent electrode. For the mapping procedure, a 128- or 192 unipolar electrode array was used (inter-electrode distances of 2.0 mm).

**Fig. 1:**
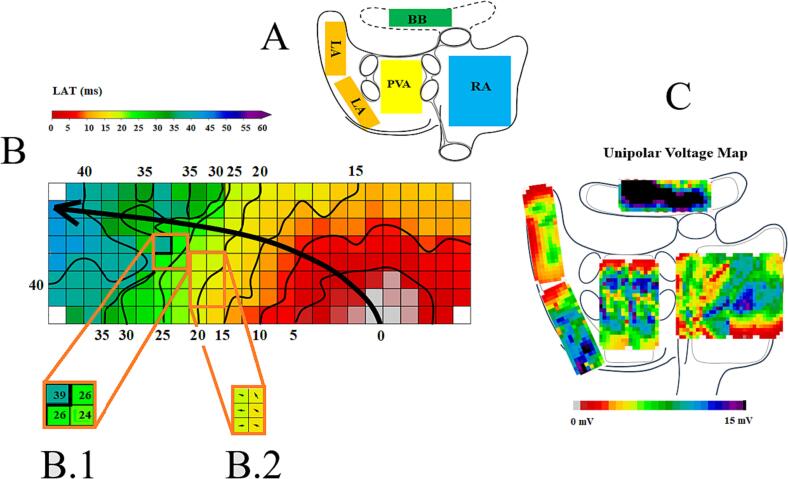
Epicardial mapping scheme, activation and voltage map. **Panel A** Epicardial mapping scheme. Panel B depicts a typical example of an activation map with isochrones and lines of CB (black lines) obtained from the RA. Panel B.1 depicts an example of CB lines (black) with an activation time difference of more than 12 ms. Panel B.2 illustrates a conduction velocity map with arrows showing the direction of activation, with larger arrows showing faster conduction velocities. Panel C shows an example of a total atrial unipolar potential voltage map (mV). BB: Bachmanns Bundle, CB: conduction block, LA: left atrium, LAT: local activation time, PVA: pulmonary vein area, RA: right atrium. Fig. 1

A predefined mapping scheme (panel A of Fig. 1) was applied for epicardial mapping, covering the entire epicardial surface of the RA, BB, LA and PVA. The RA was mapped from inferior vena cava inferior towards the superior vena cava superior in 4 consecutive horizontal positions perpendicular to the terminal crest. BB was mapped from the tip of the left atrial appendage across the roof of the LA, behind the aorta towards the superior cavo-atrial junction. Mapping of the LA was performed from the lower border of the left inferior pulmonary vein (PV) along the left atrio-ventricular groove towards the left atrial appendage and the PVA was accessed from the oblique sinus.

At every mapping site, five seconds of SR were recorded, including unipolar epicardial electrograms, a bipolar reference electrogram, a surface ECG lead and a calibration signal of 2 mV and 1000 ms. Data were stored on a hard disk after amplification (gain 1000), filtering (bandwidth 0.5–400 Hz), sampling (1 KHz) and analogue to digital conversion (16 bits).

### 2.3 Mapping data analysis

Custom-made software, previously described in detail, was used for analyzing mapping data [14], [15], [16]. By annotating the steepest negative deflection of each extracellular potential, activation maps were constructed during 5 s of SR (panel B of Fig. 1). Premature atrial complexes and aberrant beats were excluded.

Panel B of Fig. 1 demonstrates calculation of conduction times (CTs) by subtracting local activation times of each electrode from either the adjacent right or lower electrodes. Comparable to a series of prior mapping studies performed in large cohorts of patients with coronary artery disease and/or valvular heart disease, CTs ≤ 4 ms were considered as ‘normal’ intra-atrial conduction (16), CTs between 7 and 11 ms (CV of: 18–29 cm/sec) were labeled as conduction delay (CD) and CTs > 12 ms (CV: <18 cm/s) as conduction block (CB). Prevalence.

of lines of CB and cCDCB lines are expressed as a percentage of the total available number of inter-electrode connections. Areas with uninterrupted lines of CD and CB were defined as continuous CDCB lines (cCDCB). [8], [14] For both CB and continuous CD and CB (cCDCB) lines lengths were measured on a 2 mm resolution scale.

Local CV was computed as an average of velocity estimations between surrounding electrodes (longitudinal, transversal and diagonal) using discrete velocity vectors as previously described by van Schie et al (panel B.2 of Fig. 1) [17].

Unipolar voltage was defined as the peak to peak amplitude of the steepest potential deflection and unipolar potential voltages were determined at every electrode for all beats. The amount of low voltage areas (LVAs) was determined as the percentage of potentials < 1 mV for each atrial region separately (panel C of Fig. 1) [18]. Total atrial activation time (TAT) (ms) was calculated relative to the reference electrode by subtracting time differences between the earliest activated RA and latest activated LA site.

## 3 Statistical analysis

Statistical analysis was performed using the IBM SPSS Statistics 24 software. A propensity score matching analysis was performed, using logistic regression based on underlying heart disease, age, left atrial dimension and presence of obesity (BMI > 30).The (nearest) neighboring propensity score (match tolerance of 0.05) determined the randomly assignment of cases to the controls. All data were tested for normality using the Shapiro Wilk Test for normality. Normally distributed data as well as skewed data is depicted as median [interquartile range]. Normally distributed variables and skewed data were tested by using respectively a paired T-Test, and Wilcoxon Test. Dichotomous variables were examined using a McNemar test. A two-sided P-value of < 0.05 was considered statistically significant.

## 4 Results

### 4.1 Study population

Characteristics of males (N = 53, age: 65 ± 12 years) and matched females (N = 53, age: 66 ± 11 years) are summarized in Table 1. In both groups, most patients had a normal left ventricular function and non-dilated atria. Males and females only differed in the prevalence of hypertension (M: N = 25 (47,2%) versus F: N = 37 (69,8%); P = 0.031).

**Table 1:** Patient characteristics.

	**Male**	**Female**	**P- value**
	(N = 53)	(N = 53)	
**Age (years)**	65 ± 12 (21–84)	66 ± 11 (22–82)	0.219
**Risk Factors, N (%)**			
• Hypertension	25 (47,2)	37 (69,8)	0.031
• Diabetes Mellitus	14 (26,4)	22 (41,5)	0.186
• Hypercholesterolemia	17(32,1)	26 (49,0)	0.124
• BMI	27,67 ± 4,17	28,08 ± 4,74	
• Obesity	16 (30,2)	16 (30,2)	1
**Left Ventricular Function**			
• Normal (EF > 55 %)	49 (92,4)	52(98,1)	
• Moderate impairment (EF 36–45 %)	2 **(**3,8)	1 (1,9)	
• Severe impairment (EF < 35 %)	2 (3,8)	**–**	
**LA Dilatation, N (%)**	12 (22,6)	10 (18,9)	1.0
**Surgical Procedure, N (%)**			
• IHD	29 (54,7)	29 (54,7)	
• AVD	8 (15,1)	8 (15,1)	
• MVD	4 (7,5)	4 (7,5)	
• IHD + AVD	8 (15,1)	8 (15,1)	
• IHD + MVD	2 (3,8)	2 (3,8)	
• CHD	2 (3,8)	2 (3,8)	

AVD: aortic valve disease, BMI: body mass index, CHD: congenital heart disease, EF: ejection fraction, IHD: ischemic heart disease, LA: left atrium, MVD: mitral valve disease, TVD: tricuspid valve disease.

### 4.2 Mapping database

A total number of 1,031,711 (males: 503,692 versus females: 528,019) potentials were recorded. At the RA, BB, LA and PVA, a total number of respectively 464,141 (males: 234,566 versus females: 229,575), 116,466 (males: 52,044 versus females: 64,422), 196,509 (males: 94,690 versus females: 101,819) and 254,595 (males: 122,392 versus females: 132,203) potentials were recorded during 5 s of SR. When comparing males and females, males had longer SR cycle lengths (males: 855 ms [759–1013] versus females: 769 ms (693–904) (P = 0.001). Remarkably, TAT did not differ between males and females (males: 122 [76–191] ms versus females: 118 ms [40–231] ms; P > 0.05).

### 4.3 Sex differences in unipolar potential voltages

**Fig. 2:**
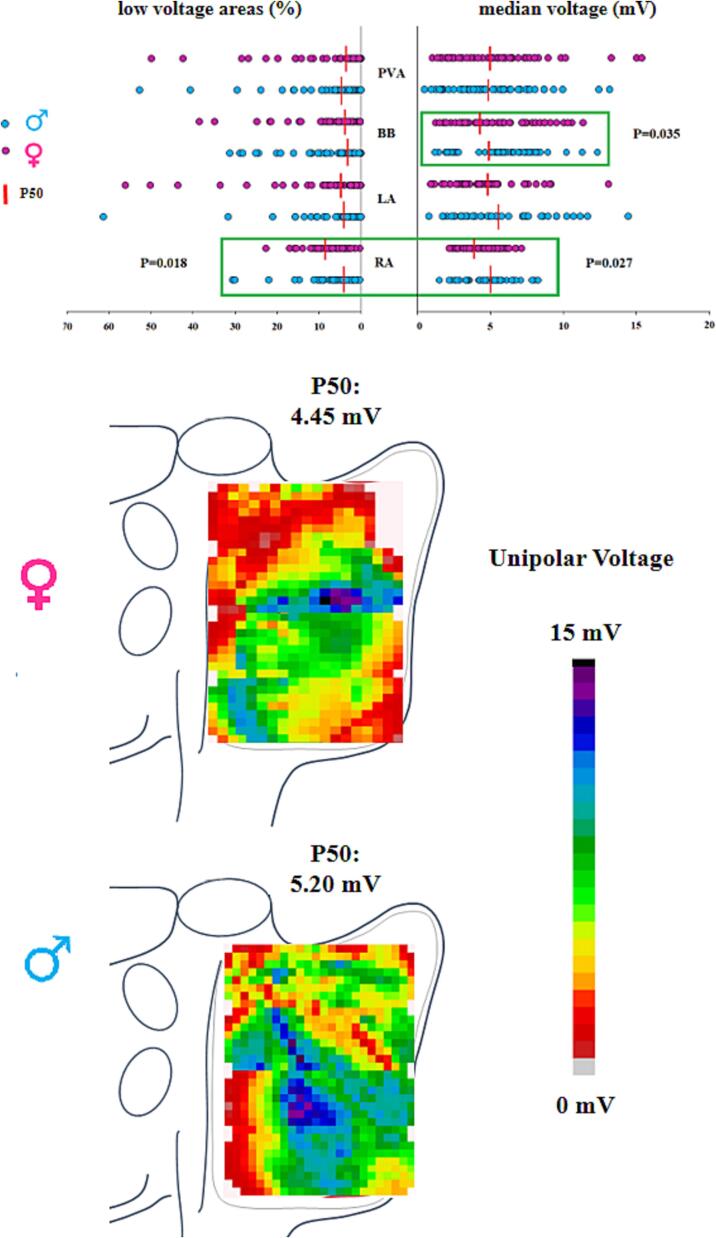
Comparison of atrial voltages between males and females. The upper panel demonstrates the amount of low voltage areas (left panel) and median unipolar potential voltages (right panel) in male and female patients for all 4 atrial regions separately. The middle and lower panel show examples of RA unipolar potential voltage maps of respectively a female and male patient. BB: Bachmanns Bundle, LA: left atrium, PVA: pulmonary vein area, RA: right atrium. Fig. 2

Unipolar potential voltages of all atrial potentials ranged from 0,5 to 17,45 mV in males and from 0,88 to 15,35 mV in females. The upper panel of Fig. 2 depicts median unipolar potential voltages and the percentage of LVAs measured at the RA, BB, LA and the PVA in males and females separately. Median unipolar potential voltages measured at the RA and BB were significantly lower in females patients (RA: males versus females: N = 234,566; 5,12 [4,22–5,94] mV versus N = 229,575; 4,39 [3,43–5,35] mV; P = 0.027, BB: males versus females: N = 52,044; 5,18 [3,23–7,79] mV versus N = 64,422; 4,4 [2,95–5,42] mV; P = 0.035) whereas unipolar potential voltages measured at the other atrial regions were comparable (P > 0.05) (Table 2).

**Table 2:** Median unipolar voltages and percentage of low voltages.

**Median Unipolar Voltages (mV)**			
	**M**	**F**	**P-Value**
**RA**	N = 234,566 5,12 [4,22–5,94]	N = 229,575 4,39 [3,43–5,35]	**0.027**
**BB**	N = 52,044 5,18 [3,23–7,79]	N = 64,422 4,4 [2,95–5,42]	**0.035**
**LA**	N = 94,690 5,86 [2,78–7,47]	N = 101,819 5,11 [3,21–7,44]	>0.05
**PVA**	N = 122,392 4,64 [3,47–6,95]	N = 132,203 5,25 (2,75–7,20]	>0.05
**Percentage of Unipolar Low Voltages (mV)**			
	**M**	**F**	**P-Value**
**RA**	4,15 [2,45–7,31]	7,19 [4,29–10,25]	**0.018**
**BB**	2,29 [0,56–9,14]	3,92 (1,7–10,36]	>0.05
**LA**	4,11 [1,53–11,0]	5,0 [2,85–9,19]	>0.05
**PVA**	4,44 [1,70–8,37]	2,91 [1,09–11,43] > 0.05	
**Conduction Velocity (cm/s)**			
	**M**	**F**	**P-Value**
**RA**	92 [86,61–95,0]	83,81 [80,10–89,72]	**0.016**
**BB**	91,15 [80,96–100,0]	86,35 [72,86–92,56]	**0.05**
**LA**	89,70 [78,43–95,59]	88,23 [79,49–94,93]	>0.05
**PVA**	98,28 [87,06–101,62]	95,09 [85,21–100,28]	>0.05
**Proportion of CB (%)**			
	**M**	**F**	**P-Value**
**RA**	2,19 [1,06–3,57]	2,8 [1,58–4,84]	**0.031**
**BB**	2,54 [1,0–6,18]	2,48 [1,40–5,43]	>0.05
**LA**	0,96 [0,30–2,26]	0,62 [0,14–1,52	>0.05
**PVA**	0,97 [0,17–1,95]	0,90 [0,29–1,87]	>0.05
**Proportion of cCDCB (%)**			
	**M**	**F**	**P-Value**
**RA**	5,50 [3,58–7,31]	6,0 [4,47–9,39]	**0.006**
**BB**	6,01 [4,29–11,46]	7,5 [5,21–9,99]	>0.05
**LA**	4,06 [2,0–5,94]	4,47 [2,16–5,62]	>0.05
**PVA**	3,60 [2,03–5,53]	4,34 [2,30–6,48]	>0.05
**Maximum Length of CB lines (mm)**			
	**M**	**F**	**P-Value**
**RA**	27 [15–36]	32 [20–42]	**0.04**
**BB**	14 [8,5–25,5]	14 [10–26]	>0.05
**LA**	14 [6–21]	9 [6–20]	>0.05
**PVA**	12 [6–23,5]	12 [7–16]	>0.05
**Maximum Length of cCDCB lines (mm)**			
	**M**	**F**	**P-Value**
**RA**	38 [27–50]	44 [29–66]	**0.05**
**BB**	24 [16–36]	30 [20–40]	>0.05
**LA**	26 [16–34]	18 [12–26]	>0.05
**PVA**	25 [16–40]	26 [18–33]	>0.05

BB: Bachmann’s Bundle, CB: conduction block, cCDCB: continuous conduction delay conduction block line, LA: left atrium, PVA: pulmonary vein area, RA: right atrium.

The upper left panel of Fig. 2 shows that LVAs were more frequently observed at the RA in females compared to males (males versus females: 4,15 [2,45–7,31] % versus 7,19 [4,29–10,25] %; P = 0.018). However, there were no significant differences between males and females in the amount of LVAs at BB, LA and PVA, (P > 0.05) (Table 2).

### 4.4 Sex differences in conduction heterogeneity

**Fig. 3:**
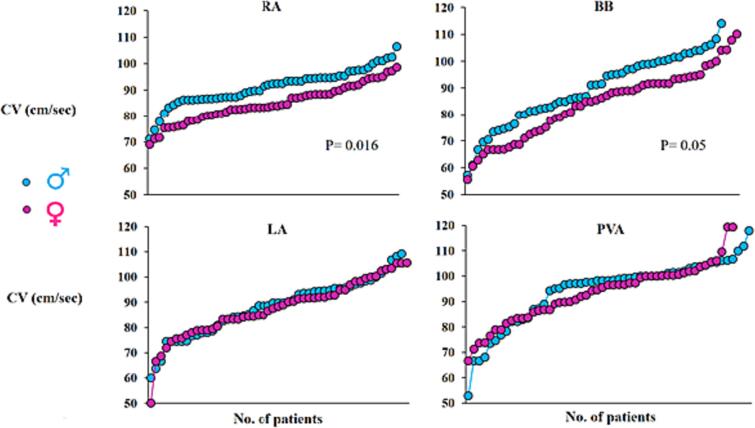
Comparison of conduction velocities between males and females. Both upper and lower panels depict the CV (cm/sec) in the entire male and female population for all atrial regions separately. BB: Bachmanns Bundle, CV: conduction velocity, LA: left atrium, PVA: pulmonary vein area, RA: right atrium. Fig. 3

As demonstrated in Fig. 3, median CV ranged from 52,84 to 117,85 cm/s in males (total number of calculated vectors: 433,092) and from 50 to 119,28 cm/s in females (total number of calculated vectors: 467,242). At both the RA and BB, median CV was significantly lower in females compared to males (RA: males versus females: 92,0 [86,61–95,0] cm/s versus 83,81 [10], [11], [12], [13], [14], [15], [16], [17], [18], [19], [20], [21], [22], [23], [24], [25], [26], [27], [28], [29], [30], [31], [32], [33] cm/s, P = 0.016 and BB: males versus females: 91,15 [80,96–100] cm/s versus 86,35 [72,86–92,56] cm/s, P = 0.05). Median CVs at all other regions were comparable (P > 0.05) (Table 2). Additionally, the relation between CVs and sinus rhythm cycle length was analyzed and no significant correlation was detected in both females and males (P = 0.38 in males and 0.89 in males). Hence, sinus rhythm cycle length does not seem to be a confounding factor.

**Fig. 4:**
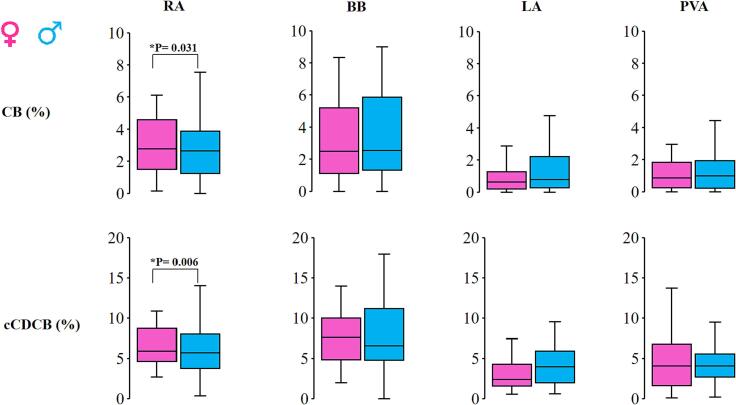
Comparison of blocklines between males and females. Boxplots showing the amount of CB (upper panel) and cCDCB (lower panel) for the male and female population separately for every atrial region. BB: Bachmanns Bundle, CB: conduction block, cCDCB: conduction block conduction delay, LA: left atrium, PVA: pulmonary vein area, RA: right atrium. Fig. 4

The proportion of CB and cCDCB lines in both males and females is shown in Fig. 4 for every atrial region separately. Only at the RA, the proportion of CB and cCDCB lines were significantly higher in females patients (males versus females: CB: 2,19 [1,06–3,57] % versus 2,78 [1,58–4,84] %; P = 0.031, cCDCB: 5,50 [3,58–7,31] % versus 6,0 [4,47–9,39]; P = 0.006). At the remaining atrial regions however, no significant differences in the number of CB or cCDCB lines were observed between females and males (P > 0.05) (Table 2).

**Fig. 5:**
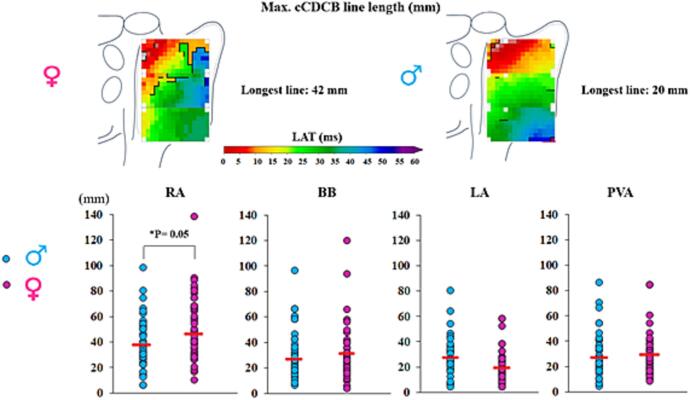
Example of an atrial activation map with lines of block and maximum line length. The upper panel depicts examples of a right atrial activation map with lines of cCDCB obtained from a male (right panel) and female (left panel) patient. The lower panels show the maximum cCBCB length (mm) for all atrial regions for every male and female patient separately. BB: Bachmanns Bundle, cCDCB: conduction delay conduction block. LA: left atrium, PVA: pulmonary vein area, RA: right atrium. Fig. 5

The upper two panels of Fig. 5 show examples of lines of cCDCB lines at the RA of a representative male and female patient. As depicted in the lower panel of Fig. 5, the maximum length of cCDCB lines was only longer at the RA in females compared to males (males versus females; CB: 27 [15–36] mm versus 32 [20–42] mm; P = 0.04 and cCDCB: 38 [27–50] mm versus 44 [29–66] mm; P = 0.05). At all other regions, there were no differences between males and females in maximum CB and cCDCB line lengths (P > 0.05, Table 2).

## 5 Discussion

### 5.1 Key findings

Differences in electrophysiological properties (during SR) of the atria between males and females were particularly found at the RA and to a lesser degree at BB. In female patients, both the RA and BB contained more areas of conduction disorders and low voltage potentials.

### 5.2 Total atrial excitation time

Reports on sex differences in atrial electrophysiological properties during SR are rare and mainly limited to analysis of P-waves on the surface electrocardiogram. It is generally assumed that the P-wave represents the total activation time of both atria. Prior studies demonstrated that P-wave durations in males are longer compared to females and that this difference remains despite age-related increase in P-wave durations [8], [19]. One possible explanation of the longer P-wave durations in males was their larger sized atria. In the present study, as described in the methodology section, TAT was calculated relative to the reference electrode by subtracting time differences between the earliest activated RA and latest activated LA site and did not differ between males and females. Given the extensive mapping of the epicardial surface of the atria, it is unlikely that parts might have been missed. The absence of shorter TAT in females may be due to extensive conduction abnormalities found in the RA in our female population which in turn prolongs TAT.

### 5.3 Sex-based differences in atrial electrophysiology

Endo- or epicardial mapping studies aimed at investigating sex-based differences in atrial electrophysiological properties are limited to the LA. Wong et al [7]. compared left atrial electro-anatomical maps constructed before pulmonary vein isolation in males and post-menopausal females of comparable ages (age 61 ± 8 years) and found that in females, CV was slower, bipolar potentials had lower voltages and were more often fractionated [7]. However, these maps were constructed during coronary sinus pacing and not during SR. During coronary sinus pacing, the RA is activated in a caudo-cranial instead of cranio-caudal direction. As a result of the non-uniform anisotropy of atrial tissue, there will be direction dependent differences in CV and potential morphology [20], [21].

Our study is the first to compare atrial electrophysiological properties between males and females during normal sinus rhythm.

In female patients, at both the RA and BB, unipolar potential voltages were lower and compared to male patients; CV at these sites was even approximately 10 cm/s lower. LVAs (<1mV) were most prounced at the RA only. In addition, the proportion and length of cCDCB lines was larger in females at this site. Lower unipolar potential voltages can be explained by a lower cardiac mass in females, yet this does not explain why these differences are confined to only RA and BB. Detailed examination of the RA anatomy using formalin preserved human hearts and cardiac magnetic resonance images (N = 100, 55 % females) demonstrated no differences in RA anatomy between males and females of comparable ages (48 ± 13 years). Hence, the observed sex differences in electrical properties in our study are most likely the result of sex differences in structural remodeling [22].

Lower potential voltages and conduction disorders may also be the result of alterations of underlying atrial tissue. e.g. by fibrotic remodeling [23], [24]. Fibrotic remodeling may be more pronounced in females as they have higher adipokine levels compared to males [25]. Adipokine causes tissue inflammation which in turn stimulates fibrotic remodeling. If so, it remains unknown why remodeling occurs preferential at the RA and BB. Likewise, slowing of conduction and lower voltage potentials at BB may also be caused by histopathological alterations. Becker et al. indeed demonstrated by microscopic studies of BB -obtained from patients (N = 10) with coronary artery and/or mitral valve disease with or without AF- that fibro-fatty replacement with a patchy distribution was clearly present in all patients. However, the size of this population was too small to investigate sex differences in fibrotic remodeling [26].

Interestingly, in our population without a history of AF, there were no sex-based differences found within the LA and PVA. These regions are assumed to play a major role in the pathophysiology of AF. It could be that when AF develops, sex-based differences in electrophysiological properties also extend towards the LA and/or the PVA.

### 5.4 Sex differences in AF triggers

Haissaguere et al.demonstrated that AF can be triggered by ectopic foci originating the pulmonary veins (PVs) and that isolation of the PVs prevents AF recurrences [27]. However, AF can also be triggered by non-pulmonary foci, such as the superior or inferior vena cava, crista terminalis or coronary sinus[28], [29]. In a multicenter observational study including 5010 patients, non-PV foci were more often ablated in the 1369 females during the index procedure compared to the males. These non-PV foci originated from the coronary sinus and intra-atrial septum [28]. In our study, areas of conduction abnormalities were also more pronounced in the RA of females. These areas of poor cell-to-cell coupling result in diminished electrotonic inhibition thereby allowing a rapidly discharging focus to become apparent [30].

## 6 Conclusion

This is the first epicardial mapping study comparing not only electrophysiological properties of LA also of BB, LA and PVA between female and male patients. Sex-based differences in conduction disorders and potential voltages were particularly found at the RA and to a lesser degree at BB. Future studies will have to show whether the more extensive arrhythmogenic substrate in female patients are related to a higher AF burden or more frequent AF recurrences after pulmonary vein isolation compared to age-matched male patients.

## 7 Clinical implications

Conduction disorders and LVAs are assumed to play a role in both initiation and perpetuation of AF. Prior mapping studies demonstrated that conduction disorders measured during SR are more pronounced in patients with AF episodes compared to patients without AF [10], [31]. This raises the question whether the more extensive conduction disorders found in female patients also indicates that they are more vulnerable to development of AF. This finding is in contrast with the observation that AF occurs more frequently in males irrespective of their age. However, several studies suggested that, despite the fact that male patients have a higher age-adjusted incidence and prevalence of AF [32], females have a higher AF burden and have more frequent AF recurrences after pulmonary vein isolation [6], [33]. As demonstrated by Wong et al., a higher amount of conduction disorders and LVAs in the LA was associated with a higher AF recurrence rate after pulmonary vein isolation. [7] Whether a more extensive potential arrhythmogenic substrate observed in female patients is also related to a higher AF burden once AF has developed needs to be proven.

## 8 Strenghts and limitations

Our mapping study not only provides data on conduction inhomogeneity of the LA, but additionally also of PVA, BB and RA in a population without a history of AF which is a strength of our study. As we performed intra-operative mapping, we only included patients who had an indication for cardiac surgery. Our findings can therefore not be translated to healthy subjects.

Acknowledgment of grant support

We did not receive financial support for completion of this manuscript.

Sources of funding

We did not receive funding for completion of this manuscript.

Registration number of clinical studies

This study was performed as part of two prospective observational projects (MEC 2010–054 and MEC 2014–393) [10,11,12]. MEC 2010–054 and MEC 2014–393). Approval of both projects were granted by the local ethics committee of the Erasmus Medical Centre and adhere to the Declaration of Helsinki principles; written consent was obtained from participating patients before the surgical intervention.

Due to the many existing collaborations within the Netherlands, we kindly ask the editor not to include reviewers from the Netherlands.

## CRediT authorship contribution statement

**Danny Veen:** Conceptualization. **Corina Schram-Serban:** Writing – review & editing. **Mathijs van Schie:** Data curation. **Frank van Schaagen:** Investigation, Review and editing. **Paul Knops:** Review and editing. **Maryam Kavousi:** Review and editing. **Yannick Taverne:** Investigation, Review and editing. **Natasja M.S. de Groot:** Conceptualization, Supervision.

## Declaration of competing interest

The authors declare that they have no known competing financial interests or personal relationships that could have appeared to influence the work reported in this paper.
